# Picture book reading on the development of preschoolers in rural areas of China: Effects on language, inhibition, and theory of mind

**DOI:** 10.3389/fpsyg.2022.1030520

**Published:** 2022-11-24

**Authors:** Yuanxia Zheng, Danyang Li, Zhongqi Chen, Guoxiong Liu

**Affiliations:** ^1^Institute of Moral Education, School of Psychology, Nanjing Normal University, Nanjing, China; ^2^NicoMama Research Center of Parent-Child Bonding, Hangzhou, China

**Keywords:** child development, rural China, book reading intervention, PPVT, inhibition, theory of mind

## Abstract

Studies have shown that book reading intervention may scaffold children's language development. However, whether book reading interventions are equally effective for children's cognitive development in a Chinese rural school setting remains to be explored. We conducted a four-month book reading intervention to address these issues in rural Chinese areas. A total of three hundred twenty-one children aged between 2.56 and 6.47 years (*M* = 4.66 ages, *SD* = 0.80) were assigned to three groups as follows: (a) control group without donated picture books; (b) active reading control group with donated picture books; and (c) intervention group with a 4-month instructed picture book reading intervention. The findings indicate that the available books could produce significant positive changes in the development of receptive language (*F*_(1,191)_ = 14.46, *p* < 0.01) and inhibitory control (*F*_(1,190)_ = 7.64, *p* = 0.01) of rural children. However, a 4-month intervention was noneffective at boosting participants' performance on these tasks (*F*_(1,203)_ = 0.07~2.73, *p* > 0.10). The results discussed the possible explanations, implications for behavioral intervention researchers, and suggestions for social service organizations or public institutions.

## Introduction

Book reading, as one of the most important educational activities (Ni et al., 2021), is closely related to child development, like problem-solving (Sajedi et al., 2018) and academic achievement (Brown et al., 2022). Many researchers have investigated book reading interventions that are based on the Vygotskian principle (Vygotsky, 1978) that social interactions with peers and parents scaffold children's development over the past 40 years (Dowdall et al., 2020). The terminology varied in different studies, like shared picture book reading interventions (Whitehurst et al., 1988; Dowdall et al., 2020), book-sharing interventions (Dowdall et al., 2020), shared book reading interventions (Chacko et al., 2018; Noble et al., 2020), and shared reading interventions (Noble et al., 2019). They all refer to the practice of reading books with the child (Noble et al., 2019), including many styles or forms, like reading themselves or with others, individually or in groups. “Sharing,” as a typical form, is applied frequently in book reading intervention studies and relates to training adults (parents, teachers, or practitioners) to read with children by using a particular style (such as interactive reading, Noble et al., 2020). We used the term “book reading intervention” because it has been frequently used in the existing literature and can represent the practice of this study that included both sharing with others and reading by themselves.

Available research has documented that book reading supports early language skill development (Fitton et al., 2018; Noble et al., 2019; Riordan et al., 2022), like vocabulary (Farrant and Zubrick, 2012, 2013; Vally et al., 2015; Marjanovič-Umek et al., 2017; Mendelsohn et al., 2020), reading ability (Silva-Maceda and Camarillo-Salazar, 2021), social communication skills (Lever and Sénéchal, 2011; Brown et al., 2018), child socioemotional development (Ni et al., 2021), attention (Cooper et al., 2014; Vally et al., 2015), IQ, and working memory (Mendelsohn et al., 2020). However, some evidence also refutes the effect of book reading intervention. For example, Noble et al. (2020) investigated interactive shared book reading interventions on children's language skills and reported that the interventions did not benefit children's language development. Some other research also showed that book reading intervention does not affect children's oral inferencing ability (Davies et al., 2020) and the complexity of language (Lever and Sénéchal, 2011). Given the inconsistent findings in the previous literature, one goal of this study was to understand the effect of book reading interventions further.

Book reading intervention research is mainly conducted in high-income countries (HICs) with well-established pediatric services (Dowdall et al., 2020), like dialogic reading (Arnold et al., 1994; Chacko et al., 2018), Parent-Child Reading Program (McElvany and Artelt, 2009), Parent-Child Home Program (Gfellner et al., 2008), Raising a Reader (Anthony et al., 2014), Reach Out and Read (Needlman et al., 2005; Klass et al., 2009), and Video Interaction Project (Cates et al., 2018). These studies have shown specific interventions' significant positive impacts on child language development. In recent years, several pieces of research have also been on book reading interventions in low and middle-income countries (LMICs). Valdez-Menchaca and Whitehurst (1992) conducted the first trial using 20 children aged 2 years from a low-income Mexican area and found significant improvement in standardized language tests. Furthermore, similar studies were conducted in rural areas of Bangladesh (Opel et al., 2009) and South Africa (Cooper et al., 2014; Vally et al., 2015; Murray et al., 2016). Wing-Yin Chow and McBride-Chang (2003), Wing-Yin Chow et al. (2008) explored the effects of book reading interventions on children of Hong Kong Chinese kindergarteners and found a positive impact on children's literacy growth and language development. *The Shenzhen Rainbow Flowers Children Readers*, the first registered grassroots nonprofit parent-child reading organization founded in 2009 in Shenzhen, examined the positive association with reading outcomes (Ni et al., 2021).

However, most of the current research is focused on parent-child interaction, that is, to improve and promote parents' book-sharing skills or encourage parents to engage in interactive reading with their children. Parents may effectively promote their children's language development; however, it may be difficult for some parents to involve in family reading activities, let alone some professional book reading intervention (Lonigan and Whitehurst, 1998). Low-income families generally have poor reading environments and less awareness of reading. The frequency of shared reading in these homes is relatively low (Adams, 1990), or even no reading activities. In China, mainly in rural areas, there are 13.84 million children in the compulsory education stage. It is almost impossible for family reading activities due to their parental absence. Most parents in low-resource families in China lack the ability and awareness to support their children's reading because of their low educational attainment (Ni et al., 2021).

Previous intervention studies in China have been conducted in urban areas (Hong Kong, Wing-Yin Chow and McBride-Chang, 2003; Wing-Yin Chow et al., 2008; Shenzhen, Ni et al., 2021). To the best of our knowledge, no studies have examined the effects of book reading interventions in rural areas, which is of great significance to theoretical understandings of the book reading interventions' products and practical enhancement of rural children's reading problems.

Almost every Chinese child aged 3 years begins their preacademic training in a kindergarten. Early exposure to reading would scaffold children's language development (Niklas et al., 2016). Kindergarten is the primary way for these rural children to be exposed to reading. Nevertheless, rural kindergartens are low-resource (e.g., they lack picture books), and teachers have low reading awareness and reading skills. Children from rural areas (low income) perform worse educational outcomes and may always stay behind (Lonigan and Whitehurst, 1998). These resource barriers raise the question of how to promote and enhance child language development in low socioeconomic status (SES) contexts. Book reading interventions are more inexpensive and can be easier to deliver than those more comprehensive interventions (Dowdall et al., 2020). Thus, this study aimed to establish the case for the scale-up of book reading interventions based on school settings in rural China.

Besides improvements in language, there are observational studies that show the positive association between book reading with children's other cognitive abilities, e.g., attention (Cooper et al., 2014; Vally et al., 2015) and working memory (Mendelsohn et al., 2020), and social cognitive performance, like theory of mind (ToM, Adrian et al., 2005; Cates and Nicolopoulou, 2019). According to the executive function (EF) theory (Diamond, 2013), inhibitory control is closely related to attention and working memory (Raver and Blair, 2016), enabling us to suppress strong internal predispositions or external lures and instead do what is more appropriate or needed (Diamond, 2013). Borella et al. (2010) found that reading performance was related to inhibitory control. However, Howard et al. (2017) investigated the effects of book reading on children's EF and suggested no inhibitory improvements. Thus, there is no clear answer to whether book reading interventions improve children's inhibitory control and ToM due to the small number of studies and the mixed findings. Accordingly, a more exploratory aim of our study was to determine whether book reading intervention also benefited inhibitory control and ToM performance.

In summary, despite a good deal of research suggesting that book reading interventions support children's cognitive development, two issues remain outstanding: whether book reading interventions are equally effective (a) for children from rural areas and (b) for a range of cognitive skills (i.e., language, inhibitory control, and ToM). We designed this study to investigate whether book reading intervention improves children's language, inhibitory control, and ToM in children from Chinese rural areas. We made some improvements to the study design based on the limitations of the existing studies. First, Noble et al. (2020) suggested that factors, like a mismatch between intervention style and natural reading style of disadvantaged contexts (e.g., less educated parents) and familiarity with reading, may affect the effectiveness of book reading interventions. Considering the rural contexts and existing intervention evidence that they are effective at boosting some cognitive skills in some samples, we assigned children to an intervention group, an active reading control group, or a control group. We donated the same picture books to the intervention and active reading control groups. In the intervention group, researchers use specific reading styles (Whitehurst et al., 1988; Noble et al., 2020) to read with children. In the active reading control group (Noble et al., 2020), children read picture books independently in their school routines. In the control group, they received the usual levels of school education.

**Figure 1:**
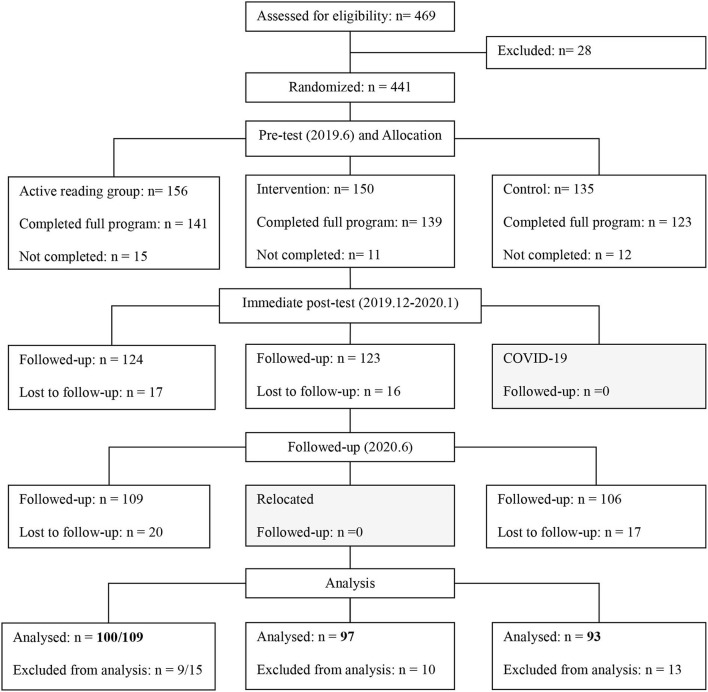
The consolidated standards of reporting trials of this study. For active reading group, *n* (T1) = 156, *n* (T2) = 124, *n* (T3) = 109; For intervention group, *n* (T1) =150, *n* (T2) =123; For control group, *n* (T1) = 135, *n* (T3) =106; In our final analyses, we compared active reading group (*n* = 109) and intervention group (*n* = 97), active reading group (*n* = 100) and control group (*n* = 93).

Second, we originally designed to conduct an immediate posttest (December 2019–January 2020) and a follow-up test (after 6 months, June 2020) on PPVT, inhibition, and ToM after our book reading intervention (refer to Figure 1). A primary limitation of the existing studies was the paucity of follow-up assessments after the immediate postintervention assessments (Dowdall et al., 2020). It is essential to examine the durability of book-sharing effects by designing a longitudinal study to establish existing long-term benefits for children's cognitive development. However, due to COVID-19 and school relocation, only the active reading control group completed three-wave tests (refer to Figure 1). Since children aged 3–5 years develop rapidly in language, inhibition, and ToM (Wellman and Liu, 2004; Diamond, 2013; Noble et al., 2019), there may be significant differences in the performance of these tasks at a 6-month interval (Adolph et al., 2008; Timmons and Preacher, 2015). We adjusted the analysis method due to unavoidable circumstances (e.g., the pandemic). We compared the active reading control group and the control group to examine the effect of the available picture books and compared the intervention group and the active reading control group to investigate the impact of book reading intervention.

Third, group-based intervention (one group included two research assistants and 15–20 children) was used in this book reading intervention. Dowdall et al. (2020) analyzed 19 studies and found that group-based interventions were more effective than one-on-one interventions. Since the teachers in these kindergartens that participated in the project did not have sufficient time and relevant psychological skills, the developmental psychology postgraduates served as experimenters for the intervention study.

Preschoolers were allocated to an intervention group, an active reading control group, or a control group. Based on the theoretical reasons for believing the effect of book reading intervention (Noble et al., 2019), we predicted that (a) children in the book reading intervention group would gain more on the language, inhibition, and ToM tests compared to those in the active reading control group and (b) children in the active reading control group would have significant gains than children in the control group. The advantage of this nonrandomized quasi-experimental study was that it allowed us to explore whether book reading boosts rural children's range of cognitive skills with higher ecological validity.

## Materials and methods

### Participants and procedure

The procedure and participants in this study are shown in Figure 1. This project lasted 1 year and 1 month between June 2019 and July 2020. The current study's participants were Chinese-speaking monolingual children recruited from three rural kindergartens in southeast China. Ethical approval for this study was received from the Nanjing Normal University. After informed consent to the children's participation was obtained from their parents or guardians, they were administered tests on language, inhibition, and ToM (June 2019, time point 1, T1). Each child was tested individually in one session lasting about 35 min at their school.

Each child's assessment and group-based intervention were conducted by either doctoral or master's students in developmental psychology. In this study, 10 postgraduates had undergone thorough training on these measurements and interventions.

Children were again administered the same tests immediately following the end of the 4-month intervention (December 2019, time point 2, T2) and ~6 months after the post-intervention session (July 2020, time point 3, T3). However, due to COVID-19 (control group, T2) and school relocation (intervention group, T3), only children in the active reading control group were followed up two times after the intervention (refer to Figure 1). There was no significant difference in children's age, *F*_(2,333)_ = 0.61, *p* = 0.54, and gender, *F*_(2,333)_ = 2.68, *p* = 0.07.

The 321 children in the final analysis (168 boys and 153 girls) ranged in age from 2.56 to 6.47 (*M* = 4.66 ages, *SD* = 0.80) at the pretest. By group, they had mean ages of 4.45 years (*SD*= 0.89, 55 boys and 42 girls) for the intervention group, 4.65 years (*SD* = 0.73, 70 boys and 61 girls) for the active control group, and 4.72 years (*SD* = 0.79, 47 boys and 46 girls) for the control group, respectively.

### Measures

#### Demographic

Demographic characteristics were assessed *via* an online questionnaire through the SurveyStar online platform (Changsha Ranxing Science and Technology, Shanghai, China) at T1. Child variables included date of birth and gender (0, girl; 1, boy). Family characteristics included family income, parents' educational level, and occupation. SES was calculated by adding the scores of family income, parents' educational level, and occupation to analyses.

#### Language

**Figure 2:**
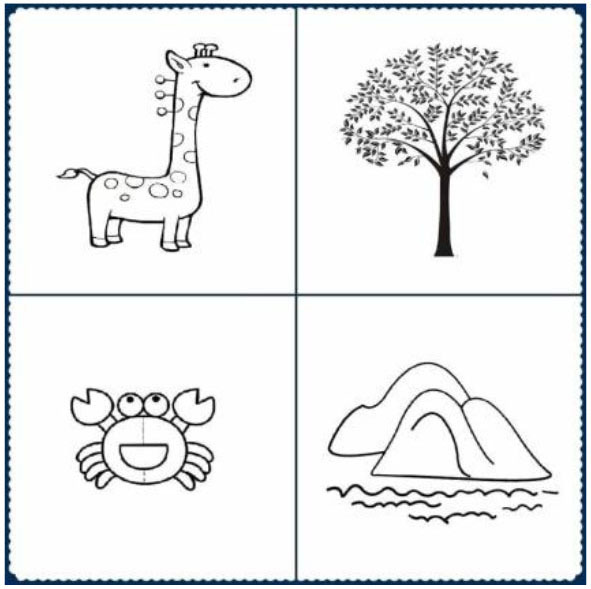
Picture materials adapted from PPVT.

Receptive vocabulary skills were measured using the Chinese version of the Peabody Picture Vocabulary Test-Revised (C-PPVT-R; Sang and Miao, 1990), which is an adaptation of the PPVT-R (Dunn and Dunn, 1981) and can be widely used to measure the vocabulary of children aged 3–9 years. DelPhi was used to compile PPVT items into a computer program and performed on a Lenovo Yoga touch-screen computer. Children were required to point to the correct one of four pictures that represents an object or action named in the instructions (e.g., “plant”, refer to Figure 2). This test contains 120 items ordered in sets of 4 pictures of the same size and similar difficulty. Each item of pictures was presented for 30 s. The participants' score was the total number of correct answers (total score: 0–120). The task was discontinued when participants made six mistakes out of eight items.

#### Inhibitory control

**Figure 3:**
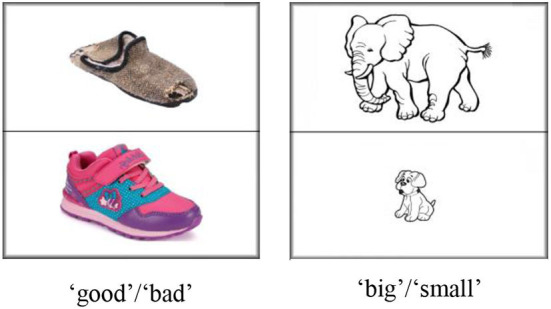
Inhibition control material. The images of shoes have been reproduced from Baidu.com.

We modified the classical Stroop paradigm (MacLeod, 1991) according to the actual situation (children were younger than reading age). In this picture–voice interference test, items were scored as correct if the child pointed to pictures that were easily recognizable and easy to understand (e.g., “good,” “big,” refer to Figure 3). The pictures and instructions were recorded and coded in the DelPhi to ensure each child received the same stimulation. The following are the main instructions:

(Congruent condition) When you hear “good,” you need to touch on the picture of “good”; when you hear “bad,” you need to touch on the picture of “bad”; (incongruent condition) when you hear “good,” you need to touch on the picture of “bad”; when you hear “bad,” you need to touch on the picture of “good.”

(Congruent condition) When you hear “big,” you need to touch on the picture of “big”; when you hear “small,” you need to touch on the picture of “small”; (incongruent condition) when you hear “big,” you need to touch on the picture of “small”; when you hear “small,” you need to touch on the picture of “big.”

Participants received four blocks of 14 trials (28 congruent and 28 incongruent trials) in a fixed order. The correct rate was used for analysis.

#### Theory of mind

Theory of mind was assessed using a set of tasks adapted from those developed by Wellman and Liu (2004), which were found to be valid and reliable for Chinese children (Wellman et al., 2006). Diverse desires *(DD)*, knowledge access *(KA)*, content false belief *(CFB)*, and explicit false belief *(EFB)* are four tasks that gradually become more complex. DD was used to assess whether a child judges that two persons (the child vs. someone else) have different desires (Wellman and Liu, 2004), including own desire question and target question. To be scored as correct, the child must answer the target question opposite to their answer to the own-desire question (ranging from 0 to 1). KA was used to assess whether a child sees what is in a container and judges the knowledge of another person who does not see what is in a container (Wellman and Liu, 2004), including the target question and the memory question. To be correct, the child must answer the target question “no” and answer the memory control question “no” (ranging from 0 to 1). CFB was used to assess whether a child judges another person's false belief about what is in a distinctive container when the child knows what it is in the container (Wellman and Liu, 2004), including two target questions and two memory questions (ranging from 0 to 2). EFB was used to assess whether a child judges how someone will search given that person's false belief (Wellman and Liu, 2004), including the target question and the reality question (ranging from 0 to 1). All tasks were acted out with figures—cartoons. This task took about 10 min.

### Intervention books

The active reading control and intervention groups were given 225 books to read during the 4-month intervention. According to the characteristics of children's cognitive and social development, two developmental psychology postgraduates screened the theme picture books, and then two child psychologists assessed whether those picture books met the children's development. We chose picture books for intervention according to children's cognitive development, e.g., *The Wolf's Chicken Stew* and *Words Are Not For Hurting*. Finally, we donated 225 picture books that were appropriate for the age group to the two groups, including 42 picture books used in the intervention.

### Intervention

Before our project, none of the three schools carried out courses related to reading picture books, and these rural children rarely touched picture books in daily life. The active reading control and intervention groups were given the same picture books. The former read books independently, and the latter was conducted to target interactive reading behaviors with two developmental psychology postgraduates during the 4-month intervention. The control condition had neither picture books nor intervention and did normal teaching activities as usual during the intervention period.

The intervention consisted of 4 h each week for four consecutive months and was delivered by 10 trained developmental psychology postgraduates. Each intervention group consisted of 15–20 children and two research assistants. Each session consisted of a reading-aloud presentation and an interactive discussion (questions and answers). The research assistants followed the style of interactive discussion devised by Whitehurst et al. (1988) that involved a series of strategies to scaffold an interactive reading between the child and adults (Noble et al., 2020). It mainly involves open questions, wh-question (i.e., who, what, where, why, when, and how), prompting every child to express. The same time was spent in the active reading control group but without specific reading interaction. In addition, the children in the intervention and active reading control groups have all the picture books available at all times.

### Analytical plan

SPSS22.0 was used for all analyses. To assess baseline differences across tasks, we used independent group *t*-tests for pretest performance. We performed a mixed 2 × 2 analysis of variance (ANOVA) in all tasks (PPVT, Stroop, and ToM) to assess differences between pretest and posttests and test for intervention effects. The ANOVA comprised time (pretest vs. posttest) and group (the active reading control vs. control; the intervention and active reading control) as factors and age as a covariate. For reporting, we started with the difference between the active reading control and control groups and reported the difference between the intervention and active reading control groups separately. All tests were two-tailed, with a significance level of *p* < 0·05. However, due to multiple comparisons, the procedure may risk inflating the family-wise alpha (Type-I) error (Ryan, 1959). The Bonferroni procedure is a classical solution to counteract the multiple comparisons problem (Wikipedia contributors, 2022): (1) the *p*-value for testing *H*_*i*_ was denoted by *p*_*i*_; (2) *H*_*i*_ is rejected if *p*_*i*_ ≤ α/*m*. *m* is the total number of hypotheses tested; in our study, *m* = 6. Thus, the *p*-value for statistical significance was adjusted to *p* < 0.005 (< 0.05/6).

## Results

### Preliminary analyses

Tables 1, 2 show the mean scores and standard deviations for three groups at the pretest and posttest and gains for all measures. There were no significant differences between the active reading control group and control group on the baseline of age (*t*_(191)_ = −0.62, *p* = 0.54), PPVT, Stroop, and a series of ToM tasks (refer to Table 1). No difference was found between the intervention group and the active reading control group on age (*t*_(204)_ = −0.16, *p* = 0.87), knowledge access, content false belief, and explicit false belief (refer to Table 2). The performance of the active control group on PPVT, Stroop, and diverse desire was significantly higher than that of the intervention group (refer to Table 2).

**Table 1:** Session performance for control and active reading control group.

	**Pretest**		**Post-test**		**Gain**	
	***M* (SD)**	***t***	***M* (SD)**	***t***	***M* (SD)**	***t***
**PPVT**						
Active reading	32.05 (15.84)	0.19	57.78 (15.66)	3.06^**^	25.73 (14.99)	3.84^***^
Control	31.56 (19.18)	49.80 (20.46)	18.24 (11.84)
**Stroop**						
Active reading	0.56 (0.23)	0.25	0.81 (0.20)	3.11^**^	0.25 (0.22)	2.77^**^
Control	0.55 (0.24)	0.70 (0.29)	0.15 (0.28)
**DD**						
Active reading	0.81 (0.39)	0.61	0.96 (0.20)	−1.28	0.15 (0.36)	−1.17
Control	0.77 (0.42)	0.99 (0.10)	0.22 (0.41)
**KA**						
Active reading	0.60 (0.45)	0.18	0.79 (0.38)	0.81	0.19 (0.47)	0.53
Control	0.59 (0.45)	0.74 (0.40)	0.16 (0.42)
**CFB**						
Active reading	0.59 (0.82)	0.44	0.95 (0.99)	−0.05	0.36 (1.11)	−0.37
Control	0.54 (0.83)	0.96 (0.98)	0.42 (1.14)
**EFB**						
Active reading	0.28 (0.45)	−1.27	0.62 (0.49)	0.56	0.34 (0.61)	1.43
Control	0.37 (0.48)	0.58 (0.50)	0.22 (0.61)

PPVT, receptive vocabulary skills; Stroop, inhibitory control; DD, diverse desires; KA, knowledge access; CFB, content false belief; EFB, explicit false belief. df = 191.

^*^p < 0.05.

** p < 0.01.

*** p < 0.001.

**Table 2:** Session performance for intervention and active reading control group.

	**Pretest**		**Post-test**		**Gain**	
	***M* (SD)**	***t***	***M* (SD)**	***t***	***M* (SD)**	***t***
**PPVT**						
Intervention	23.85 (14.20)	−2.20^*^	40.93 (19.11)	−1.49	16.02 (13.56)	−0.17
Active reading	28.29 (14.61)	44.65 (15.80)	16.36 (13.27)
**EF**						
Intervention	0.49 (0.23)	−1.97^*^	0.65 (0.30)	−1.80	0.15 (0.33)	−0.48
Active reading	0.55 (0.22)	0.72 (0.27)	0.17 (0.27)
**DD**						
Intervention	0.68 (0.47)	−3.00^**^	0.78 (0.41)	−2.09^*^	0.10 (0.34)	1.49
Active reading	0.85 (0.36)	0.89 (0.31)	0.04 (0.30)
**KA**						
Intervention	0.47 (0.44)	−1.61	0.64 (0.45)	−0.91	0.17 (0.48)	0.85
Active reading	0.58 (0.46)	0.70 (0.43)	0.11 (0.48)
**CFB**						
Intervention	0.36 (0.73)	−1.13	0.54 (0.82)	−1.94	0.16 (0.92)	−0.54
Active reading control	0.49 (0.80)	0.79 (0.94)	0.24 (1.15)
**EFB**						
Intervention	0.31 (0.46)	−0.18	0.31 (0.46)	−1.92	−0.01 (0.48)	−1.56
Active reading control	0.32 (0.47)	0.44 (0.50)	0.10 (0.50)

PPVT, receptive vocabulary skills; Stroop, inhibitory control; DD, diverse desires; KA, knowledge access; CFB, content false belief; EFB, explicit false belief. df = 204.

^*^p < 0.05.

^*^^*^p < 0.01.

^***^p < 0.001.

The SES of the active reading control group (15.34 ± 2.69) was significantly higher than the control group (13.90 ± 3.41, *t*_(148)_ = 2.87, *p* = 0.005) and higher than the intervention group (13.00 ± 3.23, *t*_(159)_ = 5.28, *p* < 0.001). SES is only significantly related to PPVT (*r* = 0.17, *p* = 0.006). Thus, we conduct further analysis without SES.

### Intervention effects

To test the effects of the intervention/language environment, we conducted several repeated-measures ANOVAs for each of the assessment tasks, with a group (intervention group and active reading control group; active reading control group and control group) as the between-subjects factor and session (pretest, posttest) as the within-subjects factor (refer to Tables 3, 4).

**Table 3:** Full statistics for the 2 (group: active reading control vs. control group) × 2 (session: pre vs. posttest) factorial ANOVAs.

	**Session**			Session*age			Session*group		
	***F***	***p*-Values**	*** $\eta_{p}^{2}$ ***	***F***	***p*-Values**	*** $\eta_{p}^{2}$ ***	***F***	***p*-Values**	*** $\eta_{p}^{2}$ ***
PPVT	17.63	0.00	0.09	0.23	0.63	0.00	14.46	0.00	0.07
Stroop	2.69	0.10	0.01	0.02	0.88	0.00	7.64	0.01	0.04
DD	19.74	0.00	0.09	11.37	0.00	0.06	1.83	0.18	0.01
KA	5.93	0.02	0.03	2.40	0.12	0.01	0.21	0.65	0.00
CFB	0.63	0.43	0.00	2.62	0.11	0.01	0.09	0.77	0.00
EFB	0.37	0.55	0.00	2.85	0.09	0.02	2.29	0.13	0.01

PPVT, receptive vocabulary skills; Stroop, inhibitory control; DD, diverse desires; KA, knowledge access; CFB, content false belief; EFB, explicit false belief.

**Table 4:** Full statistics for the 2 (intervention group vs. active reading control group) × 2 (session: pre vs. posttest) factorial ANOVAs.

	**Session**			Session*age			Session*group		
	***F***	***p*-Values**	*** $\eta_{p}^{2}$ ***	***F***	***p*-Values**	*** $\eta_{p}^{2}$ ***	***F***	***p*-Values**	*** $\eta_{p}^{2}$ ***
PPVT	0.94	0.33	0.01	2.89	0.09	0.02	0.07	0.79	0.00
Stroop	2.84	0.09	0.02	8.25	0.01	0.04	0.69	0.41	0.00
DD	1.50	0.22	0.01	3.08	0.08	0.02	2.30	0.13	0.01
KA	2.10	0.15	0.01	0.62	0.43	0.00	0.69	0.41	0.00
CFB	1.21	0.27	0.01	0.48	0.49	0.00	0.31	0.58	0.00
EFB	5.40	0.02	0.03	4.62	0.03	0.02	2.73	0.10	0.01

PPVT, receptive vocabulary skills; Stroop, inhibitory control; DD, diverse desires; KA, knowledge access; CFB, content false belief; EFB, explicit false belief.

**Figure 4:**
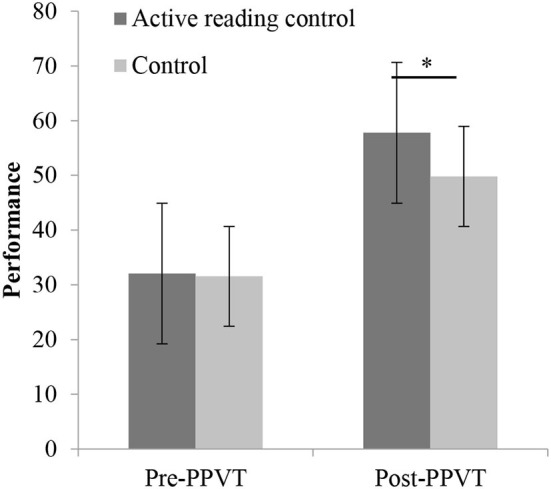
PPVT pre- and post-performance between active reading control group and control group. PPVT, receptive vocabulary skills, *p < 0.05.

**Figure 5:**
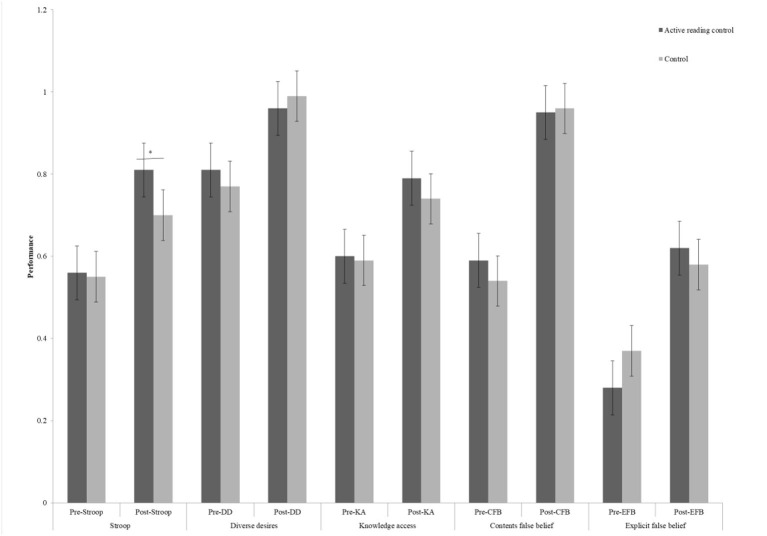
Cognitive and social-cognitive pre- and post-performance between active reading control group and control group. Stroop, inhibitory control; DD, diverse desires; KA, knowledge access; CFB, contents false belief; EFB, explicit false belief, *p < 0.05.

**Figure 6:**
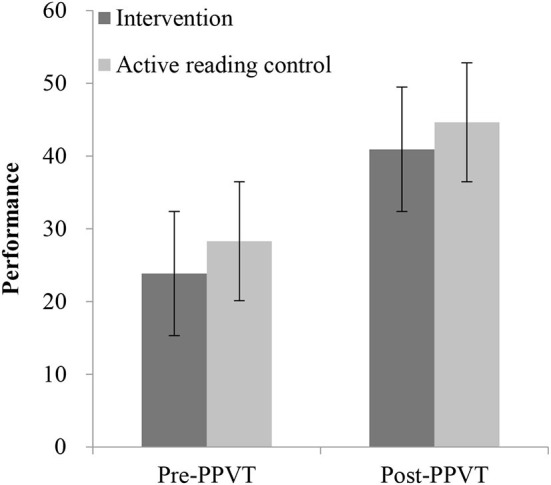
PPVT pre- and post-performance between the intervention group and active reading control group. PPVT, receptive vocabulary skills.

**Figure 7:**
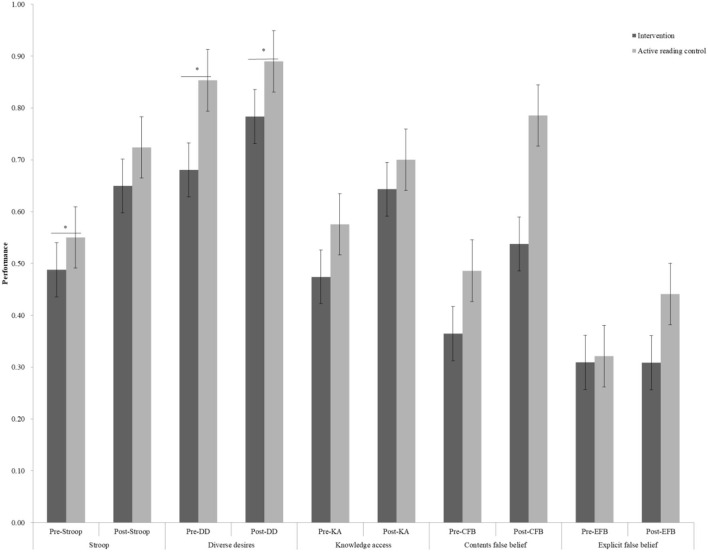
Cognitive and social-cognitive pre- and post-performance between the intervention group and active reading control group. Stroop, inhibitory control; DD, diverse desires; KA, knowledge access; CFB, contents false belief; EFB, explicit false belief, *p < 0.05.

We observed significant differences in gains for the PPVT task (*t* = 3.84, *p* < 0.01) and Stroop task (*t* = 2.77, *p* = 0.006) between the active reading control group and control group (refer to Figures 4, 5), whereas no difference could be detected for the ToM tasks (refer to Table 1). However, there was no evidence of a gain difference in all tasks between the intervention group and the active reading control group (refer to Table 2, Figures 6, 7).

Measurement of differences in performance in the PPVT task between the pretest and posttest in both active reading control group and control group reached significance, *F*_(1,190)_ = 17.63, *p* < 0.001, η^2^ = 0.09, revealing improvements from pretest to posttest in both groups. The interaction between the session and group, *F*_(1,191)_ = 14.46, *p* < 0.01, was significant. Further simple-effects analysis showed no significant difference between the two groups in the pretest, *t* = 0.19, *p* = 0.85, but there was a significant difference in the posttest, *t* = 3.06, *p* = 0.003.

For the Stroop task, results revealed the effect of the session missed significance, *F*_(1,190)_ = 2.69, *p* = 0.10, η^2^ = 0.01. The interaction between the session and group, *F*_(1,190)_ = 7.64, *p* = 0.01, η^2^ = 0.04 was significant, not reaching significance after Bonferroni adjustment. Further simple-effects analysis showed no significant difference between the active reading control group and control group in the pretest, *t* = 0.25, *p* = 0.80, but a significant difference emerged in the posttest, *t* = 3.11, *p* = 0.002.

## Discussion

This study investigated whether book reading intervention or the availability of picture books supports the development of language, inhibitory control, and ToM in children from rural China. These results demonstrate that the availability of picture books can produce significant positive changes in the development of receptive language and inhibitory control for rural children. However, contrary to our prediction, our short 4-month book reading intervention was noneffective at boosting the development of language, inhibitory control, and ToM in this sample. The following paragraphs discuss these results and their possible explanations and implications in more detail.

The difference between the active reading control and control groups indicated that the availability of picture books in low-resource educational settings could effectively promote the development of children's language and inhibitory control skills, which is an important finding. The possible explanation for why participants in the active reading control performed better would be the interval time (T1–T3, 1 year) between the pretest and posttest. From T1 to T3, participants in the active-reading control group had a whole year to read these picture books we donated before measurable improvements in language and inhibitory control could be observed. It may be practical to increase the number of picture books for rural children, as the active reading control group did. This result also provides evidence for the role of the language environment (e.g., the availability of picture books in this study) for low-resource children.

Moreover, children in the active reading control group would read picture books with their peers. This process of social interaction (Vygotsky, 1978) may scaffold their development of language and inhibitory abilities. Language development for rural children would be silently moistened during daily routines (e.g., reading picture books by themselves or with peers) rather than getting quick profits after a third-party person administering an intervention. These findings suggest that social service organizations or public institutions could provide accessible reading resources for these children from low-income areas, as we did in this study, to facilitate low-resource children's development, which may be the most convenient, economical, and efficient method.

However, the findings between the intervention group and the active reading control group indicated that our book reading intervention may lack significant effects on these measurements (i.e., PPVT, Stroop, and ToM) in this particular study. There are four possible explanations for these results. First, the standardized tests on which we measured children's performance were limited so that participants may acquire other cognitive skills not measured in this study. Consequently, our results do not necessarily generalize to other book-sharing readings. The book reading intervention may impact different language or EF skills not targeted in this study. However, our measurements are widely used in previous studies; thus, we are confident that our results have broader implications for book reading intervention. Answers to questions concerning the effects of book reading intervention for rural children seem to depend on the outcome measures.

The second possible explanation would be the combination of the intervention form (school-based) and the duration of the intervention. Our chosen 4 months were longer than some previous studies that have reported positive improvements from 1-month interventions on language development (Whitehurst et al., 1988; Opel et al., 2009). However, we only have 1 week per month to implement book reading intervention due to the academic schedule, which may affect the efficacy of the intervention. Another main difference in design lies in the intervention form, such as home-based (Whitehurst et al., 1988) and teacher-based (Opel et al., 2009). They trained parents and teachers who have the most contact with children to achieve better improvements. Child's reading outcomes are shaped mainly by family characteristics, including parental involvement and home literacy environment (Wigfield and Asher, 2002, reprint from 1984; Aikens and Barbarin, 2008). Thus, it is possible that the intervention needed the involvement of both children's teachers and parents to lead to changes in children's outcomes and needed a higher dosage (e.g., 6–12 months instead of 6–8 weeks, Noble et al., 2019).

The third explanation for why the book reading intervention did not work may be that the behaviors of book sharing did not have time to make a difference. The posttest data used for the analysis were immediate posttest, and we could not obtain follow-up data after 6 months like our research design due to the school relocation (refer to Figure 1). Perhaps participants in the intervention group need more time to implement book-sharing strategies of what they have learned before measurable improvements can be observed. Longitudinal studies are required in the future to investigate the intervention effects and the duration of the effects.

The fourth and final possible explanation for ineffective book reading interventions may be that book-sharing interactivity is no more effective than asking children to read more by themselves or their caregivers. In other words, it may be effective for rural children simply to increase the number of picture books, as the active reading control group did, without teaching children to read interactively. However, it may be premature to conclude that we dismiss all interactive book-sharing techniques based on this study alone, given that interactive book-sharing programs have been linked to children's language development in previous studies (Whitehurst et al., 1988; Wing-Yin Chow et al., 2008; Murray et al., 2016; Noble et al., 2020). Thus, we can speculate that book reading interventions can certainly impact some, but not all, children's outcomes.

Although this study provided significant evidence concerning the efficacy of book reading intervention for children from rural backgrounds and the remarkable role of the availability of picture books in the children's language and inhibitory control, some limitations of the current research should also be acknowledged. Although we designed three-wave tests in the study proposal, the regret of this study is the loss of two examinations that may provide more information for book reading intervention studies due to COVID-19 and school relocation. It implies a need for longitudinal studies with follow-up assessments after the postintervention immediate evaluations to examine the durability of book reading intervention effects. But this study gave valuable insights into how to handle modifications in research procedures due to unavoidable circumstances (e.g., pandemic). The second limitation is that we did not record the frequencies and specific performance of their reading behavior, which may affect the efficacy of the intervention (Noble et al., 2020). Future studies could detail the intervention and participants' reading behaviors. This will allow a more realistic and detailed test of the hypothesis of whether book reading interventions have a positive effect on children's outcomes.

Despite all tests and interventions provided by our research team, we still cannot control many factors in applied research settings as we can in laboratory-based research. However, compared with studies conducted under typical controlled research settings, the results of this study have higher ecological validity, i.e., they are instructive about the possible effects of related interventions or educational policies. The distinction has been referred to as efficacy experiments vs. effectiveness experiments (Lonigan et al., 1998; Bryan et al., 2021). Behavioral intervention researchers should recognize the far-reaching implications of heterogeneity and focus equitably on disadvantaged groups (like rural children) rather than just on advantaged groups that are located near research institutions or universities or high-income areas and that select samples or test sites based on convenience, which may make meaningful contributions to social progress.

## Data availability statement

The raw data supporting the conclusions of this article will be made available by the authors, without undue reservation.

## Ethics statement

The studies involving human participants were reviewed and approved by NNU20210030. Written informed consent to participate in this study was provided by the participants' legal guardian/next of kin.

## Author contributions

YZ analyzed data, wrote the paper, and wrote the first draft of the manuscript. GL, YZ, ZC, and DL performed material preparation, data collection, and analysis. All authors contributed to the study conception and design, commented on previous versions of the manuscript, read, and approved the final manuscript.

## Conflict of interest

The authors declare that the research was conducted in the absence of any commercial or financial relationships that could be construed as a potential conflict of interest.

## Publisher's note

All claims expressed in this article are solely those of the authors and do not necessarily represent those of their affiliated organizations, or those of the publisher, the editors and the reviewers. Any product that may be evaluated in this article, or claim that may be made by its manufacturer, is not guaranteed or endorsed by the publisher.
